# The effects of valve leaflet mechanics on lymphatic pumping assessed using numerical simulations

**DOI:** 10.1038/s41598-019-46669-9

**Published:** 2019-07-23

**Authors:** Huabing Li, Yumeng Mei, Nir Maimon, Timothy P. Padera, James W. Baish, Lance L. Munn

**Affiliations:** 1 Department of Material Science and Technology, Guilin University of Electronic Technology, Guilin, 541004 China; 2 Department of Radiation Oncology, Massachusetts General Hospital and Harvard Medical School, Boston, MA 02114 USA; 3 Biomedical Engineering, Bucknell University, Lewisburg, PA 17837 USA

**Keywords:** Computer modelling, Lymphatic vessels

## Abstract

The lymphatic system contains intraluminal leaflet valves that function to bias lymph flow back towards the heart. These valves are present in the collecting lymphatic vessels, which generally have lymphatic muscle cells and can spontaneously pump fluid. Recent studies have shown that the valves are open at rest, can allow some backflow, and are a source of nitric oxide (NO). To investigate how these valves function as a mechanical valve and source of vasoactive species to optimize throughput, we developed a mathematical model that explicitly includes Ca^2+^ -modulated contractions, NO production and valve structures. The 2D lattice Boltzmann model includes an initial lymphatic vessel and a collecting lymphangion embedded in a porous tissue. The lymphangion segment has mechanically-active vessel walls and is flanked by deformable valves. Vessel wall motion is passively affected by fluid pressure, while active contractions are driven by intracellular Ca^2+^ fluxes. The model reproduces NO and Ca^2+^ dynamics, valve motion and fluid drainage from tissue. We find that valve structural properties have dramatic effects on performance, and that valves with a stiffer base and flexible tips produce more stable cycling. In agreement with experimental observations, the valves are a major source of NO. Once initiated, the contractions are spontaneous and self-sustained, and the system exhibits interesting non-linear dynamics. For example, increased fluid pressure in the tissue or decreased lymph pressure at the outlet of the system produces high shear stress and high levels of NO, which inhibits contractions. On the other hand, a high outlet pressure opposes the flow, increasing the luminal pressure and the radius of the vessel, which results in strong contractions in response to mechanical stretch of the wall. We also find that the location of contraction initiation is affected by the extent of backflow through the valves.

## Introduction

Lymphatic function is critical for fluid homeostasis, and local failure leads to edema with significant morbidity and local immune dysfunction^1,2^. The collecting lymphatic vessels are responsible for pumping fluid from the tissue and delivering it to lymph nodes in order to prevent edema and maintain immune function. Although potentially beneficial for a wide variety of patients, there are currently no pharmacological modulators of lymphatic function^3–5^. The prerequisite for creating such drugs is a fundamental understanding of the mechanisms that influence lymph clearance.

The lymphatic system consists of lymphatic capillaries - termed initial lymphatics - that absorb interstitial fluid from the tissue and have unique overlapping endothelial cells^6^, which function as one -way valves to allow fluid to enter the vessels and prevent backflow into the tissue^7,8^. Initial lymphatics pass the fluid to collecting lymphatics, which contain distinct compartments defined by luminal one-way valves, known as lymphangions, that contract spontaneously to drive flow^9–12^. Thus, a collecting lymphatic vessel behaves as a group of distributed pumps aligned in series and regulated by local physiological conditions^13^. Depending on the fluid pressure conditions in the tissue and lymphatic system, collecting lymphatic vessels can adjust their behavior to maintain fluid homeostasis. Lymphatic vessels minimize contractions if existing fluid pressure gradients can drive flow, but actively pump to drive fluid otherwise^10,14,15^.

Our previous work has elucidated how these behaviors are coordinated throughout the lymphatic system by mechano-sensitive feedback^15,16^. In summary, our model shows that ${{\rm{Ca}}}^{2+}$ and NO concentrations establish complementary and oscillatory feedback loops that are self-regulating, maintaining normal lymphatic function, in agreement with experimental observations^12,17–19^. However, in our previous work, the intraluminal valves were modeled by mathematically inserting a semi-permeable wall in the vessel when the flow reversed direction. Although this approach was able to reproduce the correct behaviors seen *in vivo*, it simplified valve performance and neglected valve leaflet structure and mechanics.

The intraluminal valves that bias the flow in the direction away from the peripheral tissues are extremely important for lymphatic function, but little is known about their mechanical properties or how they influence lymph clearance or contraction efficiency. Previous studies have determined that lymphatic valves are biased to the open position, especially when the vessel is partially distended by a trans-wall pressure gradient^20^. Experimental studies and mathematical models show that this property can increase flow efficiency by reducing flow resistance when the vessel is not actively contracting, even though it results in significant backflow as the downstream lymphangion contracts^21–24^. The valve leaflets are also important as sources of biomolecules. Experiments have shown that the leaflets are a significant source of nitric oxide, presumably produced by dynamic shear stress on the leaflet structures themselves^25^.

Here, we extend our previous model of collecting lymphatic vessels by introducing realistic intraluminal valves. Furthermore, we embed the pumping vessel within a poro-elastic tissue that has fixed pressure boundaries in order to simulate fluid transport and edema at the tissue level.

## Model Description

**Figure 1:**
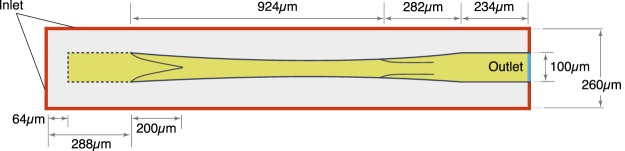
Diagram of the lymphatic vessel. *R*_0_ = 100 *μ*m is the relaxed radius of the vessel.

Figure 1 shows the model domain and dimensions. Fluid can enter the tissue from anywhere on the boundary except at the vessel outlet. Fluid percolates through the tissue to enter the initial lymphatic capillary at left (represented by the dashed line). The solid regions in this segment are impermeable, while the open regions are semipermeable, simulating the primary valves in the lymphatic capillaries. The collecting lymphatic vessel is downstream from the initial lymphatic, and fluid passes through it to exit at right. This vessel has two intraluminal valves that bias the flow toward the exit, and the walls can move to actively pump fluid. We use the lattice Boltzmann method (LBM)^26,27^ to calculate the fluid flow, shear stresses and pressures in the tissue and lymphatic vessel. The endothelium on the inner wall of the vessel can generate nitric oxide (NO)^28–30^ in response to increased shear stress, and contractions of the collecting lymphatic are determined by the concentration of ${{\rm{Ca}}}^{2+}$ in the lymphatic muscle cells. Briefly, ${{\rm{Ca}}}^{2+}$ can be depleted over time due to recharging of the cytoplasm ion concentrations, and can increase due to mechanical or chemical triggers. The self-regulating contraction dynamics that effect lymph drainage are governed by mutual mechanical feedback of the NO and ${{\rm{Ca}}}^{2+}$ systems^15,16^. The details of the model are given in the Methods section.

## Methods

### The lattice Boltzmann method

We use the lattice Boltzmann method (LBM)^26,27^ to simulate fluid flow through the tissue and lymphatic vessel. The curved boundary condition^31^ is used to keep track of the location of the vessel wall and the valves with respect to the lattice grid. The single relaxation time approximation to the Boltzmann equation can be discretized in both space and time as

1 $${f}_{i}({\bf{x}}+{{\bf{e}}}_{i},t+1)-{f}_{i}({\bf{x}},t)=-\,\frac{1}{\tau }({f}_{i}-{f}_{i}^{eq}),$$

where $\tau$ is the relaxation time. ${f}_{i}({\bf{x}},t)$ is the distribution function at time *t* and location **x**. **e**_*i*_ is the discrete velocity. ${{f}_{i}}^{eq}$ is an equilibrium distribution function that depends on fluid density and velocity. Through a Chapman-Enskog expansion, the Navier-Stokes equations can be recovered and the kinematic viscosity depends on $\tau$ as

2 $$\nu =\frac{(2\tau -1)}{6}.$$

For the D2Q9 model, one form of the equilibrium distribution is^27^

3 $${f}_{i}^{eq}={W}_{i}\rho [1+3{{\bf{e}}}_{i}\cdot {\bf{u}}+\frac{9}{2}{({{\bf{e}}}_{i}\cdot {\bf{u}})}^{2}-\frac{3}{2}{u}^{2}],$$

where, ${W}_{0}=4/9$, ${W}_{1\sim 4}=1/9$, and ${W}_{5\sim 8}=1/36$. $\rho$ and **u** are the fluid density and velocity defined by

4 $$\begin{array}{l}\rho =\sum _{i}\,{f}_{i},\\ {\bf{u}}=\sum _{i}\,{f}_{i}{{\bf{e}}}_{i}/\rho .\end{array}$$

To smooth out the hydrodynamic forces on the solid surfaces (vessel wall and valves), we use the stress -integration method. The stress tensor in the lattice Boltzmann method can be calculated as^32^

5 $${\sigma }_{ij}=-\,\frac{1}{6\tau }\rho {\delta }_{ij}-(1-\frac{1}{2\tau })\,\sum \,({e}_{\alpha i}-{u}_{i})({e}_{\alpha j}-{u}_{j}){f}_{i},$$

where ${\delta }_{ij}$ is the Kronecker delta function and $i,j=x,y$. The hydrodynamic force acting on area *S* can be calculated by integrating the stress tensor and momentum flux over *S*^32^:

6 $${\bf{F}}={\int }_{S}\{\sigma \cdot {\bf{n}}-\rho {\bf{u}}[({\bf{u}}-{\bf{V}})\cdot {\bf{n}}]\}\cdot {\rm{d}}{\bf{s}}$$

where **n** is the unit normal vector on *S* oriented towards the fluid. **V** is the velocity of *S*. In order to calculate the hydrodynamic force exerted on *S*, the distribution function *f*_*i*_ on *S* in Eq. (5) must be known, and *f*_*i*_ can generally be calculated by extrapolating from the nearby fluid nodes. Here, to simplify the calculations, we use the nearest fluid node values to estimate the distribution function on *S*. Under conditions of low Reynolds number, the resulting errors are negligible. We have previously confirmed the robustness of this model in simulations of cells and blood flow^33–36^.

### The lymphatic vessel model

The schematic diagram of a lymphatic vessel with single lymphangion defined by two valves and embedded in tissue is shown in Fig. 1. The diameter of the vessel is 100 *μ*m. Fluid from the tissue can enter the porous vessel with fixed geometry at left, which represents the initial lymphatic capillaries^7,37,38^. The solid regions in this segment are impermeable, while the open regions are semipermeable, simulating the primary valves in the lymphatic capillaries. Lymphatic capillary valves allow fluid entry but resist leakage back into the tissue, so we institute a partial bounce back boundary condition in the “open” regions of this segment^39^. We use a constant bounce back ratio $\xi =0.85$, which means that 15 percent of the fluid is allowed to leak back to the tissue when the pressure conditions favor flow in that direction. The area ratio between the semipermeable and impermeable parts is 1:1. The right end of the lymphatic vessel represents the outlet, which would be connected with additional downstream lymphangions or a lymph node. In this region, we introduce a fixed segment 234 *μ*m long to reduce the effect of the right side boundary and provide structural support.

Although many vasoactive substances can alter vessel contractility^40–42^, NO appears to play a major role in lymphatic muscle cells. NO is rapidly produced by endothelium in response to changes in shear stress and is quickly degraded^43^. For simplicity, our analysis focuses on the effects of NO on vessel contractions, but could be generalized to other flow-mediated relaxation factors. The endothelium on the inner wall of the vessel can generate nitric oxide (NO)^28–30^, which is allowed to convect and diffuse in both the fluid and tissue spaces^15^:

7 $$\begin{array}{rcl}{\rm{\Delta }}{C}_{{\rm{NO}}}({\bf{x}},t) & = & {D}_{{\rm{NO}}}{\nabla }^{2}{C}_{{\rm{NO}}}({\bf{x}},t){\rm{\Delta }}t-{\bf{u}}\cdot \nabla {C}_{{\rm{NO}}}({\bf{x}},t){\rm{\Delta }}t\\  &  & +\,(-{K}_{{\rm{NO}}}^{-}{C}_{{\rm{NO}}}({\bf{x}},t)+{K}_{{\rm{NO}}}^{+}|\frac{\partial {v}_{l}}{\partial {x}_{n}}|)\lambda {\rm{\Delta }}t,\end{array}$$

where ${C}_{{\rm{NO}}}$ is the concentration of NO. ${D}_{{\rm{NO}}}$ is the diffusion coefficient of NO. ${\nabla }^{2}{C}_{{\rm{NO}}}({\bf{x}},t)$ can be approximated using a second order finite difference scheme:

8 $${\nabla }^{2}\varphi =\sum \,\frac{{\partial }^{2}\varphi }{\partial {{x}_{i}}^{2}},\frac{{\partial }^{2}\varphi }{\partial {{x}_{i}}^{2}}\approx (\varphi ({x}_{i}+\delta )-2\varphi ({x}_{i})+\varphi ({x}_{i}-\delta ))/{\delta }^{2},$$

where $\delta =1$ is the discretized distance. ${\bf{u}}\cdot \nabla {C}_{{\rm{NO}}}$ can also be approximated using an upwind scheme

$${u}_{i}\frac{\partial \varphi }{\partial {x}_{i}}\approx \{\begin{array}{ll}{u}_{i}\frac{\varphi ({x}_{i})-\varphi ({x}_{i}-\delta )}{\delta }, & {\rm{when}}\,{u}_{i}\ge 0,\\ {u}_{i}\frac{\varphi ({x}_{i}+\delta )-\varphi ({x}_{i})}{\delta }, & {\rm{when}}\,{u}_{i} < 0.\end{array}$$

**Figure 2:**
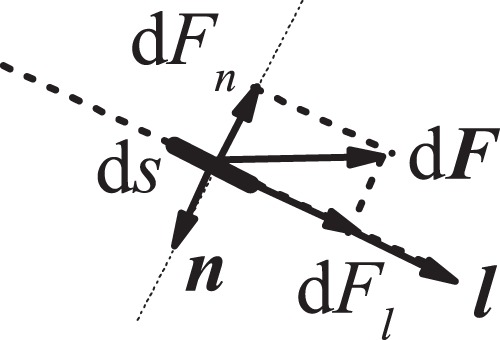
Hydrodynamic force d***F*** acting on surface element d*s*.

In Eq. (7), *λ* is the chemical reaction rate constant. $\lambda {\rm{\Delta }}t$ determines the chemical reaction time scale. Δ*t* is the lattice time scale. As *λ* increases, the chemical reaction rate increases. ${K}_{{\rm{NO}}}^{-}$ and ${K}_{{\rm{NO}}}^{+}$ are the decay rate and production coefficient of NO. Here we consider that the production of NO is proportional to the stress component $|\frac{\partial {v}_{l}}{\partial {x}_{n}}|$^16,44,45^. *v*_*l*_ indicates fluid velocity along the tangential direction of the membrane surface and *x*_*n*_ is the normal direction vector. Within the lymphangion, the valve structures can also generate NO. In fact, as shown in Fig. 2, we can indirectly calculate $\frac{{\rm{\partial }}{v}_{l}}{{\rm{\partial }}{x}_{n}}$ using the hydrodynamic force d*F*_*l*_ we calculate in Eq. (6). Thus $\frac{\partial {v}_{l}}{\partial {x}_{n}}={\rm{d}}{F}_{l}/(\rho \upsilon {\rm{d}}s)$.

Ca^2+^ enters and leaves the cytoplasm of the lymphatic muscle cells and can also pass through junctions to neighboring cells^46–49^; therefore, we use the reaction diffusion equation, with diffusion restricted to the 1D space of the vessel wall^15^:

9 $$\begin{array}{rcl}{\rm{\Delta }}{C}_{{\rm{Ca}}}({\bf{x}},t) & = & {D}_{{\rm{Ca}}}{\nabla }^{2}{C}_{{\rm{Ca}}}({\bf{x}},t){\rm{\Delta }}t\\  &  & +\,(\,-\,{K}_{{\rm{Ca}}}^{-}(1+{K}_{{\rm{Ca}},{\rm{NO}}}{C}_{{\rm{NO}}}){C}_{{\rm{Ca}}}+{K}_{{\rm{Ca}}}^{+}\\  &  & +\,{K}_{{\rm{Ca}}}^{+}{(\frac{(R-{R}_{l})}{({R}_{{\rm{Ca}}}-{R}_{l})})}^{11}+10{K}_{\delta }^{+}\delta \uparrow ({C}_{{\rm{th}}},{C}_{{\rm{Ca}}}))\lambda {\rm{\Delta }}t.\end{array}$$

Here *C*_Ca_ is the concentration of Ca^2+^. *D*_Ca_ is the effective diffusion coefficient of ${{\rm{Ca}}}^{2+}$ spreading from one cell to the neighboring cells along the vessel wall. Note that the speed of the ${{\rm{Ca}}}^{2+}$ signal in the confined space of the vessel wall is faster than 3D diffusivity, and is related to the speed of an action potential^50^. ${\nabla }^{2}{C}_{{\rm{Ca}}}$ can also be approximated through Eq. (8), but here, *δ* should be changed to a cell length of 1.51 on the lattice. ${K}_{{\rm{Ca}}}^{-}$ is the decay rate of ${{\rm{Ca}}}^{2+}$. The total decay rate of ${{\rm{Ca}}}^{2+}$ is multiplied by $(1+{K}_{{\rm{Ca}},{\rm{NO}}}{C}_{{\rm{NO}}})$ based on the fact that NO acts on the lymphatic muscle cells through myosin light chain phosphatase to reduce force generation^51–53^. ${K}_{{\rm{Ca}}}^{+}$ is the production rate of ${{\rm{Ca}}}^{2+}$. ${K}_{{\rm{Ca}}}^{+}{(\frac{(R-{R}_{l})}{({R}_{{\rm{Ca}}}-{R}_{l})})}^{11}$ determines the strength of the nonlinear elasticity of the membrane^16^. The 11th power term was arbitrarily chosen to provide a rapidly increasing force that prevents the solid elements from colliding, which would cause a logic error. Other similar functions could be substituted without significant consequence. *R* and *R*_Ca_ are the local radius of the vessel and the reference radius of the nonlinear term respectively. *R*_*l*_ is the limiting minimum radius of the vessel. $\delta \uparrow$ is an asymmetric Kronecker *δ*–function. If *C*_Ca_ increases from below to exceed the threshold *C*_th_, this function is set to 1; otherwise, it is set to 0. This is based on the fact that when *C*_Ca_ reaches the threshold *C*_th_, it triggers action potentials mediated by voltage gated and calcium-induced calcium channels^16,54^. ${K}_{\delta }^{+}$ defines a steep increase of *C*_Ca_ while ${C}_{{\rm{Ca}}} > {C}_{{\rm{th}}}$.

There are five forces that act on the wall of the vessel:

1. The hydrodynamic force *F*, which is calculated by stress integration using the LBM. In order to calculate *F*, the walls of the vessel are discretized into *N* segments, and the segments can only move along the *y* direction.
2. The lymphatic muscle force depends on the concentrations of ${{\rm{Ca}}}^{2+}$ and NO according to^15^:
  10 $${F}_{M}={K}_{M}(\frac{{C}_{{\rm{Ca}}}}{1+{C}_{{\rm{Ca}}}})(\frac{2R}{R+{R}_{{\rm{Ca}}}})(\frac{1}{1+{K}_{{\rm{NO}}}{C}_{{\rm{NO}}}}),$$
  where ${K}_{M}$ is the coefficient determining the strength of action.
3. Elastic force from the tissue:
  11 $${F}_{E}=-\,{K}_{E}(R-{R}_{0}).$$
  In order to limit the contraction amplitude and reproduce tissue mechanical resistance, if $R < {R}_{l}+{\rm{\Delta }}$, we simply increase *F*_*E*_ by multiplying an 11th power item. Eq. (11) changes to:
  $${F}_{E}=-\,{K}_{E}(R-{R}_{0})\ast {(\frac{{\rm{\Delta }}}{R-{R}_{l}})}^{11},\,{\rm{while}}\,R < {R}_{l}+{\rm{\Delta }}.$$
4. Bending force:
  12 $${F}_{B}=-\,{K}_{B}((y-{y}_{m})+(y-{y}_{n})).$$
  Here we consider that the left and right sides of the vessel wall are fixed; in between, the wall can move along the y-direction. ${y}_{m}$ and ${y}_{n}$ are the y-direction positions of the neighboring points of the vessel.
5. Considering the vessel is viscoelastic, a viscous resistance force is introduced as:

13 $${F}_{r}=-\,{K}_{r}v.$$

The minus sign means that ${F}_{r}$ always acts in the direction opposing wall velocity $v$.

**Figure 3:**
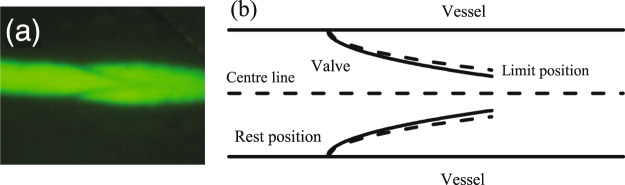
Simulating intraluminal lymphatic valves. (**a**) Intravital image of a lymphatic valve in the popliteal collecting lymphatic of a mouse. (**b**) Parabolic valve shape assumed for the model.

As shown in Fig. 3, the bileaflet valve (illustrated in Fig. 3(b)) has structure similar to that of a real lymphatic valve (Fig. 3(a))^55,56^. The valve is treated as two opposing deformable parabolic - shaped membranes. Compared with previous models of valves^57–60^, our 2D valve model has been simplified to provide computational efficiency for the multi-lymphangion simulations while, at the same time, recapitulating the relevant physics, biology, physiology and biochemistry of the lymphatic vessel.

The membrane also has elastic force ${f}_{E}^{v}$, bending force ${f}_{B}^{v}$ and viscous force ${f}_{r}^{v}$ shown in Eqs (11–13). The rest position of lymphatic valves has been shown to be biased in the open position^22,23^. Thus, we set our valve position to a parabolic line (solid line in Fig. 3(b)), the equation of which is

14 $${y}_{0}=H\pm \sqrt{(x-{x}_{0})/B},$$

where ${y}_{0}$ indicates the rest or limit position of the valve. $H$ is the rest position of the vessel, ${x}_{0}$ is the anchor point of the valve. ‘−’ and ‘+’ indicate upper and lower valve leaflets. $B$ can adjust the rest position to be biased to stay open or close. For the limit position, $B$ is maximum. In the open configuration, the ${f}_{E}^{v}$ and ${f}_{B}^{v}$ of the valve are designated

$$\begin{array}{rcl}{f}_{E}^{v} & = & -{k}_{E}^{v}(y-{y}_{0}),\\ {f}_{B}^{v} & = & -{k}_{B}^{v}(2y-y(i+1)-y(i-1)-(2{y}_{0}-{y}_{0}(i+1)-{y}_{0}(i-1))),\end{array}$$

where *i* indicates a discrete segment of the valve. When the two membranes of a valve come into close proximity, we also increase ${f}_{E}^{v}$ by multiplying an 11th power term:

15 $${f}_{E}^{v}=-\,{k}_{E}^{v}(y-{y}_{0}){(\frac{{\rm{\Delta }}}{y-{y}_{c}})}^{11},\,{\rm{when}}\,{y}_{c} < y\le {y}_{c}+{\rm{\Delta }},$$

where ${y}_{c}$ is the center line of the vessel (dash line in Fig. 3(b)). When the segment arrives at the limit position *y*_*l*_ shown as in Fig. 3(b), we also increase ${f}_{E}^{v}$ as:

16 $${f}_{E}^{v}=-{k}_{E}^{v}(y-{y}_{0}){(\frac{{\rm{\Delta }}}{{y}_{l}-y})}^{11},\,{\rm{w}}{\rm{h}}{\rm{e}}{\rm{n}}\,({y}_{l}-{\rm{\Delta }})\le y < {y}_{l}.$$

**Figure 4:**
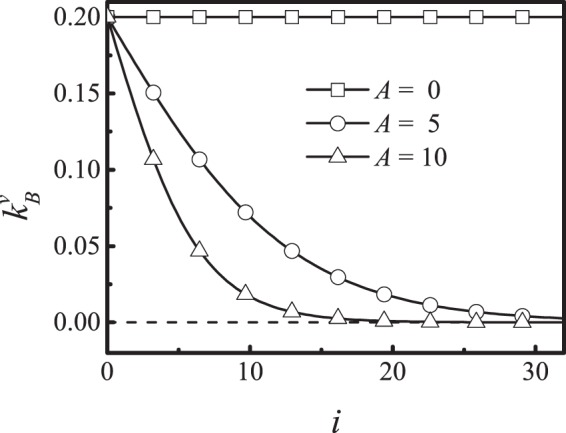
Bending modulus of the valve decreases from the anchor point to the free end.

The valve mechanical properties have evolved to ensure proper valve operation. The leaflets must be flexible, so that they can form an effective seal, but rigid enough to resist the hydrodynamic forces as the valve closes during backflow. Therefore, we assume the valve mechanical properties vary along the length. Although this assumption has not yet been verified for these small lymphatic valves, mathematical models and experiments have shown that large valves in veins have such spatially- variable stiffness^60^ (see Fig. 3(b)). To satisfy both these requirements, we specify that the leaflets have variable rigidity by dividing them into segments: near the attachment region at the wall, the leaflets are stiff, but they become more flexible at the tip. Specifically, the varying bending coefficient is set according to:

17 $${k}_{B}^{v}=\frac{2({k}_{0}^{v}-{k}_{R}^{v})}{1+\exp (Ai/n)}+{k}_{R}^{v},n\ge i\ge 0,$$

where $i$ indicates segment number and $n$ is the total number of segments. A membrane of the valve is discretized from left to right into $n$ segments. ${k}_{0}^{v}$ is the maximum of ${k}_{B}^{v}$. ${k}_{R}^{v}$ is an approximation of the minimum of ${k}_{B}^{v}$, the coefficient $A$ adjusts the soft range of the valve. Figure 4 shows how ${k}_{B}^{v}$ changes over the length of the valve leaflet.

To ensure that the total inner force is zero, Eq. (12) changes to:

18 $$\begin{array}{rcl}{F}_{B}^{v} & = & -{k}_{B}^{v}(i-1)(y-y(i-1)-({y}_{0}-{y}_{0}(i-1)))\\  &  & -\,{k}_{B}^{v}(i)(y-y(i+1)-({y}_{0}-{y}_{0}(i+1))).\end{array}$$

There are special considerations as the valve leaflets close. If we specify that the valve can close completely, then there will be no unoccupied lattice points on the center line of the vessel, and the two membranes could approach without being separated by any fluid nodes. We avoid the consequent computational difficulties and unrealistic fluid dynamics by introducing a lubrication force that establishes the hydrodynamic force in this region (see “Lubrication force” below). When the membranes of the valve approach the center line of the vessel, we change the rest position of the valve to a straight line and the bending force is changed to:

19 $$\begin{array}{rcl}{F}_{B}^{v} & = & -{k}_{B}^{v}(i-1)(y-y(i-1))\\  &  & -\,{k}_{B}^{v}(i)(y-y(i+1)).\end{array}$$

For motion of the valves and vessel wall, we assume the structures have the same viscosity. The vessel and the valves move according Newton’s laws of motion. At each Newtonian dynamics time step, the center of mass of each segment is updated by a so-called half-step ‘leap-frog’ scheme^61^:

20 $${\bf{V}}(t+\frac{1}{2}\delta t)={\bf{V}}(t-\frac{1}{2}\delta t)+\delta t{\bf{a}}(t),$$

21 $${\bf{X}}(t+\delta t)={\bf{X}}(t)+\delta t{\bf{V}}(t-\frac{1}{2})+\delta {t}^{2}{\bf{a}}(t),$$

where **V** is the velocity of the center of mass of each segment, *δt* is the Newtonian time step, here chosen to be $\delta t=1/100$ lattice time step, and **a** is the acceleration of each segment. When the segments move, new fluid nodes are revealed; we extrapolate the status of each new fluid node based on the known quantities of those fluid nodes located on the same side of the moving boundary.

### Lubrication force

**Figure 5:**
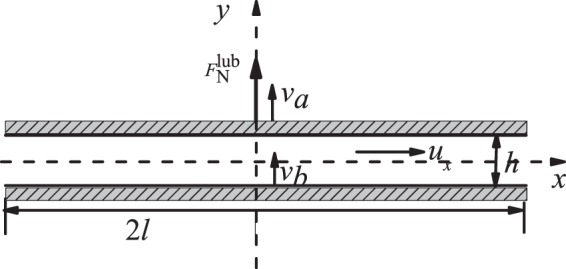
Schematic of lubrication force between to planes.

When two membranes approach each other, they can displace all the fluid, and the hydrodynamic force cannot be calculated using LBM. In this case, we use lubrication theory to calculate the hydrodynamic force^62^. As shown in Fig. 5, we only consider the normal lubrication force ${F}_{N}^{lub}$ (which acts in the y direction, because valve segments move only in the y direction) to determine the force on the valves.

According to lubrication theory^63^, the Stokes equation can be represented by

22 $$\frac{{\rm{d}}p}{{\rm{d}}x}={\eta }_{s}\frac{{\partial }^{2}{u}_{x}}{\partial {y}^{2}},$$

where *p* is the pressure, ${\eta }_{s}$ is the dynamic viscosity, and ${u}_{x}$ is the fluid velocity along the x-direction. The boundary conditions are

$${u}_{x}=\{\begin{array}{ll}0, & {\rm{when}}\,y=\frac{h}{2},\\ 0, & \mathrm{when}\,\,y=-\,\frac{h}{2}.\end{array}$$

Integrating Eq. (22) of *y*

23 $$\frac{1}{2}\frac{{\rm{d}}p}{{\rm{d}}x}({y}^{2}-\frac{1}{4}{h}^{2})={\eta }_{s}{u}_{x}+{\eta }_{s}{c}_{1}\frac{1}{h}y-\frac{{c}_{2}{\eta }_{s}}{2},$$

where:

$$\begin{array}{l}{c}_{1}=0,\\ {c}_{2}=0.\end{array}$$

From Eq. (23)

24 $${u}_{x}=\frac{1}{2{\eta }_{s}}\frac{{\rm{d}}p}{{\rm{d}}x}({y}^{2}-\frac{1}{4}{h}^{2}).$$

The flux is:

25 $$Q={\int }_{-\frac{h}{2}}^{\frac{h}{2}}\,{u}_{x}\,{\rm{d}}y=-\,\frac{1}{12{\eta }_{s}}\frac{{\rm{d}}p}{{\rm{d}}x}{h}^{3}.$$

The fluid volume between two planes in Fig. 5 is

$${V}_{b}=hx.$$

During virtual variation of *δt*, the variation of *h*_0_ is:

26 $$\delta {h}_{0}=({v}_{a}-{v}_{b})\delta t,$$

then the variation of the volume is:

27 $$\delta {V}_{b}=({v}_{a}-{v}_{b})x\delta t.$$

The flux caused by the relative movement in the normal direction is:

28 $$Q=-\,\frac{\partial {V}_{b}(t)}{\partial t}+{Q^{\prime} }_{0}({u}_{f})=-\,({v}_{a}-{v}_{b})x+{Q^{\prime} }_{0}({u}_{f}).$$

The flux ${Q^{\prime} }_{0}({u}_{f})$ is caused by flow from upstream. Because Eq. (22) is linear and the relationship between *Q* and *u*_*x*_ is also linear, the general flux can be written as:

29 $$Q=-\,({v}_{a}-{v}_{b})x+{Q}_{0}=-\,\frac{1}{12{\eta }_{s}}\frac{{\rm{d}}p}{{\rm{d}}x}{h}^{3}.$$

30 $$\frac{{\rm{d}}p}{{\rm{d}}x}=12{\eta }_{s}({v}_{a}-{v}_{b})x/{h}^{3}-\frac{12{\eta }_{s}}{{h}^{3}}{Q}_{0}.$$

31 $$\frac{{\rm{d}}p}{{\rm{d}}x}y={\eta }_{s}\frac{\partial {u}_{x}}{\partial y},$$

32 $$\frac{\partial {u}_{x}}{\partial y}=[12{\eta }_{s}({v}_{a}-{v}_{b})x/{h}^{3}-\frac{12{\eta }_{s}}{{h}^{3}}{Q}_{0}]y.$$

Because ${u}_{x}\gg {u}_{y}$, $\frac{\partial {u}_{x}}{\partial y}\gg \frac{\partial {u}_{x}}{\partial x}$, the Rayleigh’s dissipation function is^64^:

33 $$\begin{array}{rcl} {\mathcal R}  & = & \frac{1}{2}{\eta }_{s}\,{\int }_{-\infty }^{\infty }\,{\rm{d}}x\,{\int }_{-\frac{h}{2}}^{\frac{h}{2}}\,{(\frac{\partial {u}_{x}}{\partial y})}^{2}{\rm{d}}y\\  & = & \frac{1}{2}{\eta }_{s}[4{({v}_{a}-{v}_{b})}^{2}\frac{{l}^{3}}{{h}^{3}}-12\frac{({v}_{a}-{v}_{b}){Q}_{0}}{{h}^{3}}{l}^{2}+\frac{12{Q}_{0}^{2}}{{h}^{3}}l],\end{array}$$

then the normal lubrication force is:

34 $${F}_{N}^{lub}=-\,2\frac{\partial  {\mathcal R} }{\partial {v}_{a}}=-\,8{\eta }_{s}\frac{({v}_{a}-{v}_{b}){l}^{3}}{{h}^{3}}.$$

Let ${v}_{b}=-\,{v}_{a}$ (upper and lower leaflets of the valve are symmetric):

35 $${F}_{N}^{lub}=-\,16\rho \frac{2\tau -1}{6}{v}_{a}\frac{{l}^{3}}{{h}^{3}}.$$

For two planes constructed with *n* connected segments, each having length *l*, if each segment moves with the same velocity, the lubrication force exerted on an individual segment should be:

36 $${F}_{N}^{lub}=-\,16\rho \frac{2\tau -1}{6}{v}_{a}\frac{{l}^{3}}{{h}^{3}}{n}^{3}/n=-\,8\rho \frac{2\tau -1}{3}{n}^{2}{v}_{a}\frac{{l}^{3}}{{h}^{3}}.$$

If some segments move up (${v}_{y}\ge 0$) and others move down (${v}_{y} < 0$), for example, ${n}_{1}$ segments move up and ${n}_{2}$ segments move down, the lubrication force exerted on segment ${s}_{i}$ can be estimated as:

37 $${F}_{N}^{lub}=\{\begin{array}{ll}-8\rho (2\tau -1)/3{({n}_{1})}^{2}{v}_{a}\frac{{l}^{3}}{{h}^{3}}, & {\rm{if}}\,{\rm{the}}\,{\rm{segment}}\,{\rm{moves}}\,\mathrm{up},\\ -8\rho (2\tau -1)/3{({n}_{2})}^{2}{v}_{a}\frac{{l}^{3}}{{h}^{3}}, & {\rm{if}}\,{\rm{the}}\,{\rm{segment}}\,{\rm{moves}}\,{\rm{down}}{\rm{.}}\end{array}$$

The simulation domain is 433 × 66 lattice points. The calculations are performed using an NVIDIA Quadro M5000 graphics card with 2048 Cuda cores. The number of parallel threads depends on the calculations being performed. For example, for the calculations of fluid node density with LBM, we send 28578 threads to Cuda, which distributes them amongst 2048 cores. Host memory is allocated to the GPU to avoid excessive data transfer between the CPU and GPU. Codes were written in C++.

### Simulation parameters

The system time scale depends on the fluid kinematic viscosity and diameter of the vessel as: $T=\frac{\nu }{\nu ^{\prime} }{(\frac{D^{\prime} }{D})}^{2}$, where $\nu =(2\tau -1)/6$, $D=2{R}_{0}$ and $\nu ^{\prime}$, *D*′ are the actual fluid kinematic viscosity and diameter, respectively. *T* is the duration of one lattice step. The space scale depends on $L=\frac{D^{\prime} }{D}$, where *L* is the length of one lattice unit. Here we choose $\tau =0.75$, $\nu ^{\prime} =0.01\,c{m}^{2}/s$, $D=25$ and $D^{\prime} =0.01\,cm$. Thus, real time and space scale are $T=1.33\times {10}^{-6}\,s$ and $L=0.0004\,cm$. The chemical reaction speed depends on *λT*. In our simulation, we choose the simulation parameters as given in Table 1.

**Table 1:** Chemical parameters of NO and Ca^2+^; Mechanical parameters of the fluid, vessel wall and valves.

Parameter	Definition	Value	Units	Source
**FLUID**				
*L*	Lattice space unit	4	*μ*m	
*T*	Lattice time step	1.33 × 10^−6^	s	
${\rho }_{{\rm{in}}}$	Fluid density at inlet	1	g/cm^3^	As water
$\nu ^{\prime}$	Fluid viscosity	0.01	cm^2^/s	As water
$\tau$	Relaxation time	0.75	*T*	
**Chemical properties of NO and Ca** ^**2+**^				
*D*_NO_	NO diffusivity	1.2 × 10^−4^	cm^2^/s	$3.0\times {10}^{-5}\,({{\rm{cm}}}^{2}/{\rm{s}})$ ^75^
${K}_{{\rm{NO}}}^{-}$	NO degradation rate constant	3.7594	${s}^{-1}$	Estimated
${K}_{{\rm{NO}}}^{+}$	NO production rate constant	400	Dimensionless	Estimated
${D}_{{\rm{Ca}}}$	${{\rm{Ca}}}^{2+}$ signal propagation rate	$6.5\times {10}^{-6}$	$c{m}^{2}/s$	Estimated
${K}_{{\rm{Ca}}}^{-}$	${{\rm{Ca}}}^{2+}$ degradation rate constant	37.6	${s}^{-1}$	Estimated
${K}_{{\rm{Ca}}}^{+}$	${{\rm{Ca}}}^{2+}$ production rate constant	1.2	${s}^{-1}$	Estimated
${K}_{\delta }^{+}$	${{\rm{Ca}}}^{2+}$ production rate constant	15038	${s}^{-1}$	Estimated
${C}_{{\rm{th}}}$	${{\rm{Ca}}}^{2+}$ threshold	0.015	Dimensionless	Estimated
${R}_{{\rm{Ca}}}$	Threshold radius for ${{\rm{Ca}}}^{2+}$ channel sensitization	${R}_{0}$		Estimated
${K}_{{\rm{Ca}},{\rm{NO}}}$	Rate constant for NO inhibition of ${{\rm{Ca}}}^{2+}$	5.3	Dimensionless	Estimated
*λ*	Chemical reaction rate constant	0.03	Dimensionless	Estimated
**VESSEL**				
${K}_{M}$	Force constant for ${{\rm{Ca}}}^{2+}$	$7.6\times {10}^{-5}$	${\rm{dynes}}$	Estimated
${K}_{E}$	Elastic modulus the vessel	4.52	${\rm{dynes}}/{{\rm{cm}}}^{2}$	Estimated
${K}_{B}$	Bending modulus the vessel	9045	${\rm{dynes}}/{{\rm{cm}}}^{2}$	${10}^{6}\,({\rm{dynes}}/{{\rm{cm}}}^{2})$ ^66^
${K}_{r}$	Viscosity coefficient of vessel	$4.8\times {10}^{-9}$	${\rm{dynes}}\cdot {\rm{s}}/{\rm{cm}}$	Estimated
${K}_{{\rm{NO}}}$	NO inhibition of force	1	Dimensionless	Estimated
${R}_{l}$	Limit radius	0.003	*cm*	40% contraction^20^
${R}_{0}$	Rest radius of the vessel	0.005	*cm*	
**VALVE**				
$A$	How soft the valve is	5	Dimensionless	Estimated
$B$	How much the valve biased to open	1500	$c{m}^{-1}$	Estimated
${k}_{E}^{v}$	Elastic modulus of valves	9.0 × 10^−4^	${\rm{dynes}}/{{\rm{cm}}}^{2}$	Estimated
${k}_{0}^{v}$	Bending modulus of the base of valves	18090	${\rm{dynes}}/{{\rm{cm}}}^{2}$	${10}^{6}\,({\rm{dynes}}/{{\rm{cm}}}^{2})$ ^66^
${k}_{R}^{v}$	Bending modulus of the tip of valves	0.0091	${\rm{dynes}}/{{\rm{cm}}}^{2}$	$0.1{k}_{0}^{v}$ ^60^
**VESSEL & VALVE**				
Δ		2 × 10^−4^	cm	Estimated

Parameter values were estimated in order to yield physiologically reasonable results when independently measured values were unavailable.

To simulate the different masses of the vessel wall and valve leaflets, we specify different densities for these structures on the lattice. Specifically, the density of the vessel wall is assumed to be 80 times that of the valve leaflet. This incorporates both the increased cellularity of the wall vs. leaflet, but also the structural anchoring of the wall to the surrounding tissue. Initially, all the fluid node densities are set to 1, the velocities are set to 0, and the NO concentration is set to 0. The initial ${{\rm{Ca}}}^{2+}$ concentration within the vessel wall is set to 0.0149, which is close to, but less than, ${C}_{{\rm{th}}}$. The density at the boundary (red area in Fig. 1) is kept constant as ${\rho }_{in}$ by imposing an equilibrium distribution with a velocity of 0, and the density at the outlet is kept constant as ${\rho }_{out}$ through a constant pressure boundary condition^65^. NO concentration on the boundary is held constant at 0. NO can diffuse through the fluid, valve structures and vessel wall, but ${{\rm{Ca}}}^{2+}$ distributes only within the vessel wall. At the left and right sides of the domain, the calcium is treated similar to a bounce-back boundary condition. In our simulation, we keep ${\rho }_{in}=1$. Since the fluid is treated as water, the pressure unit is $P={(L/T)}^{2}\,{\rm{g}}\cdot {{\rm{cm}}}^{-3}=9.045\times {10}^{4}\,{\rm{g}}\cdot {{\rm{cm}}}^{-1}\cdot {{\rm{s}}}^{-2}$, thus the inlet pressure is held at $3.015\times {10}^{4}\,{\rm{g}}\cdot {{\rm{cm}}}^{-1}\cdot {{\rm{s}}}^{-2}$. The upper and lower vessel walls are discretized into 200 segments; the length of each is 6.03 *μ*m. Each valve is discretized into 32 segments; the length of each is 6.25 *μ*m. The Young’s modulus unit is $E={L}^{2}/{T}^{2}{\rm{g}}\cdot {{\rm{cm}}}^{-3}$. For the bending rigidity, the shear modulus is *K*d*l*, where d*l* is length of each segment of vessel or valve. Thus, the shear modulus of the vessel or valve can be calculated as $1.51{K}_{B}E$ and $1.56{k}_{0}^{v}E$, giving 13658 and 28266 dynes/cm^2^ respectively. Here 1.51 and 1.56 are the dimensionless lengths of each segment of vessel and valve on the lattice. These values for the moduli are less than those estimated for valves in large veins^66^, which are on the order of 10^6^ dynes/cm^2^, but are reasonable, given the much smaller size of our valves. Table 1 lists the model parameter values and their origin.

## Results

### The effect of valve mechanical parameters *A* and *B* on pumping

**Figure 6:**
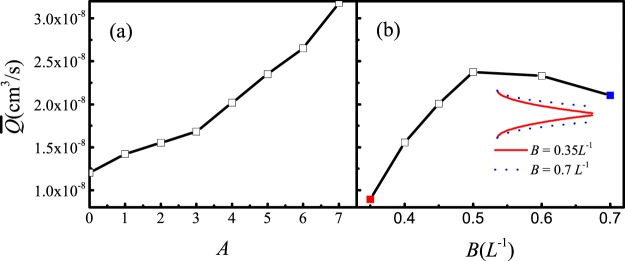
Pumping flux is affected by the mechanical properties and rest state of the valves. (**a**) Output flux is affected by the rigidity parameter *A* (defined in Fig. 4, increasing *A* means increased flexibility of the leaflet tip). Flux increases as the tip of the leaflet becomes more flexible, relative to the base. (**b**) Output flux is increased when the valve rest position is more open (Keeping $A=0.5$, increasing *B* of Eq. (14)). The inset figure shows how *B* affects the rest position of the valve.

We first investigated how valve mechanical properties affect lymph clearance and pumping behavior under equal pressure at inlet and outlet. To do this, we set the bounce back condition of the porous part of the initial lymphatic region (Fig. 1, dashed line) to allow more leakage. Setting the bounceback ratio to zero means that fluid can leave the vessel as easily as it enters. This puts more burden on the intralumenal valves to control backflow. We then varied the mechanical properties of the valve leaflets and measured performance. We first varied the parameter A, which describes the spatial dependence on bending modulus along the length of the leaflet. When *A* is zero, the tip section of the leaflet is as rigid as the base; increasing A makes the tip more flexible than the base (Eq. 17 and Fig. 4). When the rest position condition is set at $B=0.6{L}^{-1}$, increasing *A* results in a continuous increase in output (Fig. 6(a)). When A is small, the valve is too rigid, and does not close easily, resulting in excessive backflow that limits the flux through the vessel. As *A* increases, the free end of the valve becomes more flexible, and the valve can deform to resist the backflow. In Fig. 6(b), we keep $A=5$, and increase *B* from 0.35*L*^−1^ (valve closed at rest) to 0.7*L*^−1^ (valve open at rest, see inset figure). Larger values of *B* mean that the valve is more open at rest. Increasing B causes the cycle-averaged flux to increase before 0.5. This implies that, before 0.5, valves biased to stay open may allow some leakage, hurting efficiency, but this is more than offset by the decrease in overall flow resistance presented by the open valves. Above 0.6, the leakage significantly decreases the efficiency, resulting in decreased flux. This is consistent with previous models and experimental observations, which show that lymphatic valves are biased toward the open position^20,22–24^. In the remaining simulations, we set *A* = 5, *B* = 0.6*L*^−1^.

### Dynamics of Ca^2+^ concentration

**Figure 7:**
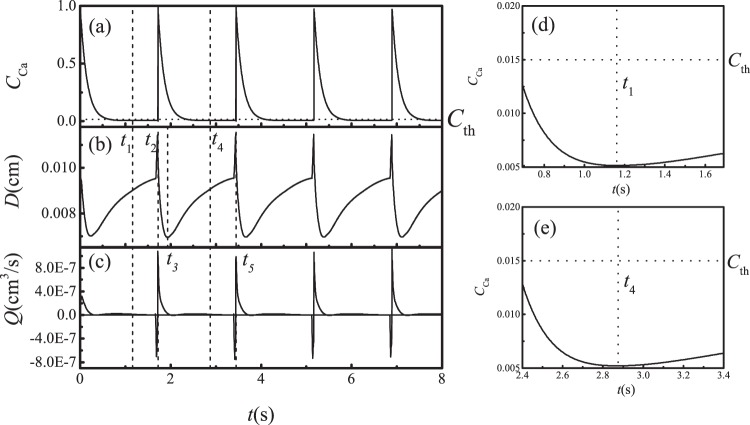
The ${{\rm{Ca}}}^{2+}$ concentration, diameter of the vessel and flux during periodic contractions, ${\rm{\Delta }}p=0.3015\,{\rm{g}}\cdot {{\rm{cm}}}^{-1}\cdot {{\rm{s}}}^{-2}$. (**a**) ${{\rm{Ca}}}^{2+}$ concentration, (**b**) diameter, and (**c**) flux. (**d** and **e**) are the enlarged curve of ${{\rm{Ca}}}^{2+}$ concentration in (**a**). The horizontal dotted line indicates the threshold of ${{\rm{Ca}}}^{2+}$ concentration.

**Figure 8:**
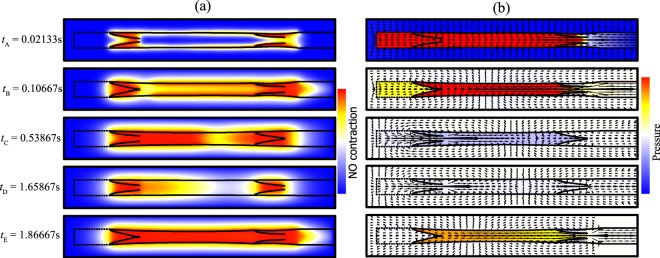
(**a**) NO concentration, (**b**) pressure and velocity field dynamics during a contraction cycle, ${\rm{\Delta }}p=0.3015\,{\rm{g}}\cdot {{\rm{cm}}}^{-1}\cdot {{\rm{s}}}^{-2}$. The minimum is blue and maximum is red. The arrows lengths are proportional to the local fluid velocity. See also Supplemental Videos S1 and S2.

Figure 7 shows how Ca^2+^ concentration, vessel diameter and fluid flux change with time when the vessel undergoes sustained spontaneous contractions with ${\rho }_{out}=0.99999$; in this case, the pressure difference between the tissue boundary and lymphangion outlet is ${\rm{\Delta }}p=0.3015\,{\rm{g}}\cdot {{\rm{cm}}}^{-1}\cdot {{\rm{s}}}^{-2}$. The changes in ${{\rm{Ca}}}^{2+}$ concentration agree with previous reports using one-dimensional models^16^. In Eq. (9), the equilibrium of ${C}_{{\rm{Ca}}}$ can be estimated as ${K}_{{\rm{Ca}}}^{+}(1+{(\frac{(R-{R}_{l})}{({R}_{{\rm{Ca}}}-{R}_{l})})}^{11})/{K}_{{\rm{Ca}}}^{-}$, thus we choose ${K}_{{\rm{Ca}}}^{+} < {K}_{{\rm{Ca}}}^{-}$ and the equilibrium of ${C}_{{\rm{Ca}}}$ is less than 1. Meanwhile ${K}_{{\rm{Ca}}}^{+}$ is large enough to ensure that the equilibrium ${C}_{{\rm{Ca}}}$ (when ${C}_{{\rm{NO}}}=0$) is larger than the threshold ${C}_{{\rm{th}}}$. Initially, ${C}_{{\rm{NO}}}=0$, and  ${C}_{{\rm{Ca}}}$ increases from the initial value to the equilibrium value. When ${C}_{{\rm{Ca}}}$ becomes larger than ${C}_{{\rm{th}}}$ (dotted line in Fig. 7(a)), in equation (9), $\delta \uparrow$ will be 1, and ${C}_{{\rm{Ca}}}$ increases steeply. The ${{\rm{Ca}}}^{2+}$ concentration reaches saturation and stops increasing; consequently, $\delta \uparrow$ becomes 0. Meanwhile, the vessel begins to contract and drives fluid downstream. This increases the shear stress on the vessel downstream. In response, the endothelial cells on the inner vessel wall and on the both surfaces of the valve leaflet generate NO that appears in the nearby fluid nodes. The NO concentration ${C}_{{\rm{NO}}}$ at the inner fluid node near the vessel and the valve downstream increases (see Fig. 8), and can reach saturation (value = 1). Because of the second term of Eq. (9), large values of ${C}_{{\rm{NO}}}$ cause ${C}_{{\rm{Ca}}}$ to decrease and the vessel begins to relax and pull in fluid, generating fluid flow upstream. As a result, the upstream fluid node near the inner surface of the vessel and both surfaces of the valve leaflet increase their generation of NO. When the ${C}_{{\rm{Ca}}}$ level recedes below the threshold ${C}_{{\rm{th}}}$, the NO concentration also decreases to the baseline. After ${C}_{{\rm{Ca}}}$ reaches the minimum, decreased NO allows ${C}_{{\rm{Ca}}}$ to increase again, starting another cycle. Because of the rapid diffusion of ${{\rm{Ca}}}^{2+}$ between neighboring cells, the movements of adjacent vessel segments are coordinated.

### Vessel contractions and fluid flow

Figure 7(b) shows how the diameter at the midpoint of the lymphangion changes with time. When ${p}_{in} > {p}_{out}$, fluid flow is driven by the hydrodynamic pressure; in this condition, because of the Venturi effect, the radius of the vessel can be less than *R*_0_. In Fig. 7(b), from ${t}_{1}$ to ${t}_{2}$, after the previous cycle of relaxation, NO reaches the baseline, and ${C}_{{\rm{Ca}}}$ begins to increase (dashed curve in Fig. 7(a)). When $t={t}_{2}$, ${C}_{{\rm{Ca}}}$ just passes through the threshold, the ${{\rm{Ca}}}^{2+}$ concentration in the wall suddenly increases and a contraction begins. When $t={t}_{3}$, the radius of the vessel reaches its minimum; the duration of the contraction is ${T}_{c}={t}_{3}-{t}_{2}=0.216s$. After the contraction, the relaxation stage begins and the ${{\rm{Ca}}}^{2+}$ concentration returns to its minimum value at $t={t}_{4}$. Because of the inertia of the system the vessel relaxes to its maximum radius at $t={t}_{5}$. The relaxation time ${T}_{r}={t}_{5}-{t}_{3}=1.509s$, and the total period of the contraction cycle is $T=1.725s$. *T*_*c*_ can be adjusted by the rate constant ${K}_{{\rm{Ca}},{\rm{NO}}}$, while *T*_*r*_ can be adjusted by ${K}_{{\rm{Ca}}}^{+}$, ${K}_{{\rm{NO}}}^{-}$ and ${D}_{{\rm{NO}}}$. *h* controls the chemical reaction speed – that is, it can adjust *T*.

Figure 7(c) shows the fluid flux averaged across the vessel lumen. The large positive flux peak is preceded by a brief negative flux at the onset of contraction. This is because the right side of the vessel contracts first due to the lower NO concentration in this region: because the fluid flows in from the left (during the relaxation phase ${t}_{3} < t < {t}_{5}$ in Fig. 7(b)), more NO is produced in that part of the vessel, and this delays the contraction in the left hand side relative to the right (see Fig. 8(a), $t={t}_{4}$). As a result, the higher ${{\rm{Ca}}}^{2+}$ on the right side initiates the contraction there. The fluid is then pushed to the left briefly (at the reference point of the vessel midpoint, which is plotted in Fig. 7(c)). The backflow is also evident in the velocity vectors, which are directed to the left when a valve is closing (Fig. 8(b), $t={t}_{1}$).This behavior has been reported by other researchers^22,67^.

### Distribution of NO, pressure and deformation of the vessel and valve

As the vessel begins to contract, the left valve closes and the right valve opens, and the resulting changes in wall shear stresses affect NO production (Fig. 8(a)). The movement of the valve further increases the shear stress on both surfaces of the leaflet, generating high levels of NO near the valves. (see Fig. 8(a), $t={t}_{A}$). Because of the backflow, some NO flows out from the left valve (Fig. 8(a), $t={t}_{B}$). The high concentration of NO exits from the opened right valve, as is evident from the convex shape of the NO gradient surrounding the right valve (Fig. 8(a), $t={t}_{B}$) (see also Fig. 8(b) for flow velocities). The higher concentrations of NO near the valves predicted by the simulations have been observed experimentally in lymphatic vessels in rats^25^.

During lymphangion relaxation, (Fig. 8(a), $t={t}_{C},{t}_{D}$), the left valve opens and the right valve is closed. Fluid flows into the vessel from the left, and more NO is generated in this region compared with the right side. After relaxation, the concentration of NO remains higher. As the vessel reaches peak diastole, (Fig. 8(a), $t={t}_{D}$), the fluid velocity becomes small, and little NO is generated. NO rapidly degrades or diffuses, and its concentration drops. Meanwhile, the ${{\rm{Ca}}}^{2+}$ concentration is lower than the threshold, and the vessel is primed for another contraction. The contraction produces high shear stresses and generates NO, which floods the vessel (Fig. 8(a), $t={t}_{E}$).

Because of the smaller effective vessel diameter between the valve leaflets, the shear stresses are largest at the valves (Fig. 8(b), $t={t}_{A}$). This, and the fact that both surfaces of the leaflet can produce NO, is responsible for the higher generation of NO shown in Fig. 8(a). In Fig. 8(a), $t={t}_{C}$, the vessel is relaxing and the left valve is open, resulting in fluid flowing in from the left side. Thus, the left side generates more NO than the right side. In Fig. 8(a), $t={t}_{D}$, the fluid almost fills the vessel and the fluid velocity becomes small everywhere, resulting in low NO generation. Meanwhile the ${{\rm{Ca}}}^{2+}$ concentration is lower than the threshold and is primed for another contraction. During the contraction, NO is generated, and it diffuses and convects, filling the vessel (Fig. 8(a), $t={t}_{E}$). In the initial lymphatic segment at left, the porous wall with simulated check valves allow some lymph leakage back to the interstitium (in the junction regions, 85 percent of leakage is reflected, while 15 percent passes).

Figure 8(b) also shows the pressure color map corresponding to Fig. 8(a). Unlike NO, fluid can not pass though the vessel wall or the valve leaflet. Thus, there are pressure discontinuities across these structures. As the vessel contracts, the pressure inside is higher than outside (Fig. 8(b), $t={t}_{A}$). In the initial lymphatic segment at left, the biased valves in the wall maintain the pressure higher than the surrounding tissue. As the contraction ends, the pressure inside the vessel decreases (Fig. 8(b), $t={t}_{B}$). As the intraluminal pressure reaches a minimum (Fig. 8(b), $t={t}_{C}$), flow enters from the left. The decreasing pressure difference across the valve leaflet in Fig. 8, $t={t}_{D}$, causes the valve to return to its rest position. Because of the small pressure difference between the inlet and outlet, the fluid velocity is very small, and the pressure is almost homogeneous, implying that the vessel almost completely relaxes. As another contraction starts, the left valve closes and the lymphangion pressure increases again (Fig. 8(b), $t={t}_{E}$). Retrograde flow at a valve can occur either from a contraction in the lymphangion(s) downstream, or by relaxation of the lymphangion(s) upstream from the valve. In Fig. 8(c) we can see a rapid backflow due to the contraction starting at the downstream region of the lymphangion. In this case, the ${{\rm{Ca}}}^{2+}$ spreads rapidly from downstream to the upstream. This is in contrast to the situation when the backflow is caused by dilation of the upstream vessel^67^, which results in slower backflow, and is also seen in our simulations when the outlet valve is closing (see Fig. 8(b), $t={t}_{C}$, between the right valve and outlet).

### Pumping cycle is affected by the pressure difference between the tissue and the lymphangion outlet

Our simulations predict that lymphatic contractions depend on NO concentration dynamics, which depend on the changing fluid stresses. Thus, adjusting the pressure drop from the tissue to the lymphatic can affect  the fluid velocity and the pumping behavior. To see how the lymphangion responds to changes in tissue pressure that might be encountered *in vivo*, we performed simulations with step changes in pressure drop. Holding the inlet density $\rho =1.0\,{\rm{g}}\cdot {{\rm{cm}}}^{-3}$, we varied the outlet density as follows:

38 $${\rho }_{out}=\{\begin{array}{ll}1.0\,{\rm{g}}\cdot {{\rm{cm}}}^{-3}, & {\rm{when}}\,0\le t < {T}_{1},\\ 0.9998\,{\rm{g}}\cdot {{\rm{cm}}}^{-3}, & {\rm{when}}\,T1\le t < T2,\\ 1.0002\,{\rm{g}}\cdot {{\rm{cm}}}^{-3}, & {\rm{when}}\,T2\le t < T3,\end{array}$$

Thus the pressure difference is

39 $${\rm{\Delta }}p=\{\begin{array}{ll}0.0, & {\rm{when}}\,0\le t < {T}_{1},\\ 6.032\,{\rm{g}}\cdot {{\rm{cm}}}^{-1}\cdot {{\rm{s}}}^{-2}, & {\rm{when}}\,T1\le t < T2,\\ -6.032\,{\rm{g}}\cdot {{\rm{cm}}}^{-1}\cdot {{\rm{s}}}^{-2}, & \mathrm{when}\,\,T2\le t < T3.\end{array}$$

**Figure 9:**
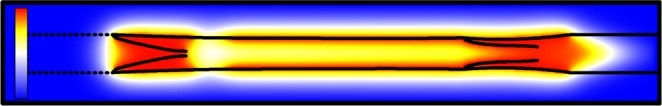
Backflow through the gap between the leaflets influences NO distribution.

To reproduce valve performance characteristics reported for actual lymphatic vessels^68,69^, we allow some backflow through the valves. We introduce an elastic force when the distance between the two valve leaflets is smaller than $2{y^{\prime} }_{0}$ as:

40 $${f}_{l}=-\,{k}_{l}(y^{\prime} -{y^{\prime} }_{0}),$$

where *y*′ is the distance between the leaflet and the vessel center line. 2*y*′ is the distance between two leaflets. Here we choose ${y{\rm{^{\prime} }}}_{0}=2$ lattice units and ${k}_{l}=0.0004$. As shown in Fig. 9, when the left side valve tries to close, this force will maintain a small gap.

**Figure 10:**
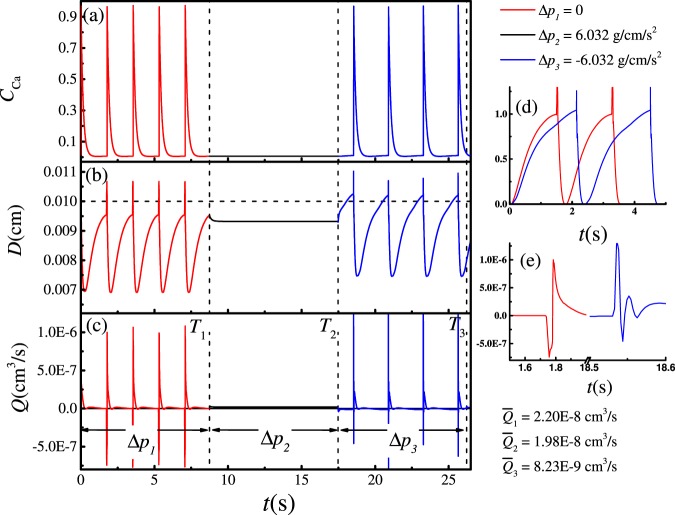
Pumping state changes with pressure difference ${\rm{\Delta }}{p}_{1}=0$, ${\rm{\Delta }}{p}_{2}=6.032\,{\rm{g}}\cdot {{\rm{cm}}}^{-1}\cdot {{\rm{s}}}^{-2}$, ${\rm{\Delta }}{p}_{3}=-\,6.032\,{\rm{g}}\cdot {{\rm{cm}}}^{-1}\cdot {{\rm{s}}}^{-2}$. (**a**) Concentration of ${{\rm{Ca}}}^{2+}$, (**b**) diameter near the outlet of the lymphangion, (**c**) flux changing with time, and (**d**) shows the plots in (**b**) for $t < {T}_{1}$ and ${T}_{2}\le t < {T}_{3}$, shifted to the same baseline time and diameter to show the differences in period and amplitude. (**e**) Expanded view of the plots in (**c**), showing details of the waveforms. ${\bar{Q}}_{1}$, ${\bar{Q}}_{2}$, ${\bar{Q}}_{3}$ are the average flux for ${\rm{\Delta }}p={\rm{\Delta }}{p}_{1}$, Δ*p*_2_, Δ*p*_3_, respectively. See also Supplemental Video S3.

**Figure 11:**
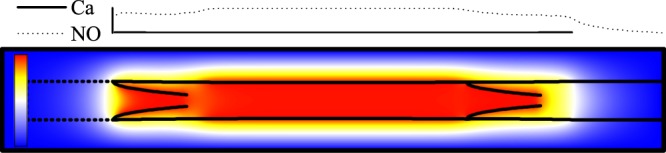
NO distribution in the relaxed state. (${\rm{\Delta }}{p}_{2}=6.032\,{\rm{g}}\cdot {{\rm{cm}}}^{-1}\cdot {{\rm{s}}}^{-2}$).

**Figure 12:**
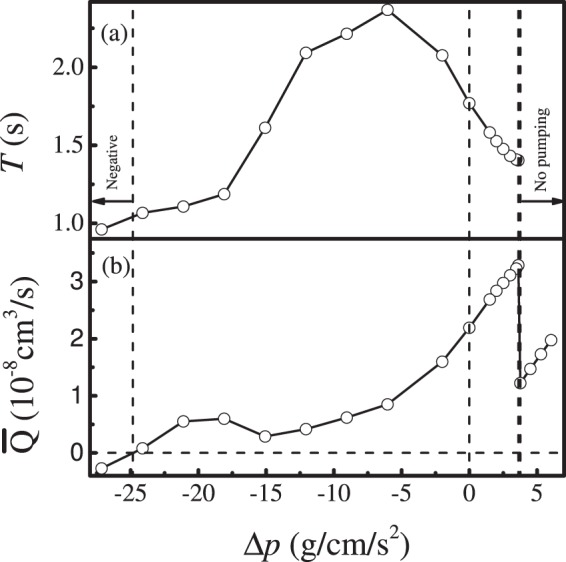
Pumping cycle and average fluxes are affected by Δ*p*.

The ${{\rm{Ca}}}^{2+}$ dynamics are shown in Fig. 10(a). As expected, in the range of $0\le t < {T}_{1}$, ${\rm{\Delta }}{p}_{1}=0$, the periodic contractions are self-sustaining. In the range of ${T}_{1}\le t < {T}_{2}$, the forward pressure difference is ${\rm{\Delta }}{p}_{2}=6.032\,{\rm{g}}\cdot {{\rm{cm}}}^{-1}\cdot {{\rm{s}}}^{-2}$. In this case, the pressure drives flow and increases the shear stress on the endothelial surfaces to generate high levels of NO. As in Fig. 11, high concentrations of NO depress ${{\rm{Ca}}}^{2+}$ levels below the threshold, and the lymphangion stops contracting (see Fig. 10(b)). The radius of the vessel is smaller than the rest radius ($R < {R}_{0}$), implying that the pressure inside the vessel is lower than outside. In the range of ${T}_{2}\le t < {T}_{3}$, the forward pressure difference changes to ${\rm{\Delta }}{p}_{3}=-\,6.032\,{\rm{g}}\cdot {{\rm{cm}}}^{-1}\cdot {{\rm{s}}}^{-2}$, meaning that the outlet pressure is higher than inlet. Because of backflow, The high pressure forces some fluid back into the vessel and expands the vessel. When the diameter of the vessel exceeds *R*_0_, the nonlinear term in Eq. (7) begins to activate, creating high levels of ${{\rm{Ca}}}^{2+}$ and making ${C}_{{\rm{Ca}}} > {C}_{{\rm{th}}}$. This triggers the next contraction (see Fig. 10(a,b)) which strongly forces fluid out of the lymphangion (see Fig. 10(d)). Meanwhile backflow generates NO, which delays ${{\rm{C}}}_{{\rm{Ca}}}$ from reaching the threshold. Thus the contraction phase is extended as in Fig. 12(a).

Figure 10(c) shows how flux is affected by the pressure difference. When ${\rm{\Delta }}{p}_{1}=0$, the vessel always starts to contract from the right side, as in Fig. 7(c), resulting in a negative spike of flux. When ${\rm{\Delta }}{p}_{2}=6.032\,{\rm{g}}\cdot {{\rm{cm}}}^{-1}\cdot {{\rm{s}}}^{-2}$, the vessel does not contract, resulting in a constant flux. When ${\rm{\Delta }}{p}_{3}=-\,6.032\,{\rm{g}}\cdot {{\rm{cm}}}^{-1}\cdot {{\rm{s}}}^{-2}$, the outlet pressure is higher than inlet. The vessel expands beyond *R*_0_ and contracts strongly. In this case, the backflow through the right valve during relaxation is sufficient to increase the concentration of NO in this region; therefore, the vessel starts to contract from the left side, resulting in a positive peak of flux at the beginning of the contraction (see Fig. 10(e)). The average flow rate values $\bar{Q}$ in Fig. 10 show that an opposing pressure gradient decreases overall flow rate because of the backflow caused by the high pressure at the outlet (see Fig. 10(c), ${T}_{2}\le t < {T}_{3}$). Perhaps the most important parameter to reproduce in the simulations is the wall shear rate, which depends on the vessel diameter as well as the fluid velocity. Examining the data in Fig. 10, our shear rate values are comparable to measurements made non-invasively *in vivo*^67,70^: the flow velocity range *in vivo* was approximately −100–600 *μ*m/s, in a ~34 *μ*m vessel, which translates to wall shear rates of −23.5 to 141.2 s^−1^. Our simulations in Fig. 10 fall within this same range, with the wall shear rate varying in the range ~−1.4 to 140.0 s^−1^.

Analyzing the total output from the vessel, averaged over time, we identify a number of regimes where the vessel exhibits interesting behaviors (Fig. 12):

1. ${\rm{\Delta }}p < -\,25\,g\cdot c{m}^{-1}\cdot {s}^{-2}$: When the opposing pressure is this high, the vessel tries to pump, but the flow is retrograde, driven by pressure through the leaky valves.
2. $25\,g\cdot c{m}^{-1}\cdot {s}^{-2} < {\rm{\Delta }}p < 0$: At more moderate opposing pressures, the vessel contracts to drive flow, and the output, in general, increases as the opposing pressure decreases. The local maximum in flow at ${\rm{\Delta }}p\approx -\,19\,g\cdot c{m}^{-1}\cdot {s}^{-2}$, is due to the generation of NO from backflow at the outlet valve; this NO inhibits contractions near the outlet, and encourages them to start near the inlet. These pseudo-peristaltic contractions tend to increase efficiency slightly. The peak in the period at ${\rm{\Delta }}p\approx -\,6\,g\cdot c{m}^{-1}\cdot {s}^{-2}$, is where NO has a large effect on the calcium dynamics, depressing Ca^2+^ far below the threshold level and thus delaying the next contraction.
3. $0 > {\rm{\Delta }}p > 3.62\,g\cdot c{m}^{-1}\cdot {s}^{-2}$: When the pressure gradient is favorable for flow (i.e., the inlet pressure is higher than the outlet), a combination of pressure-driven flow and contractions can move the fluid. Increasing pressure causes a linear increase in flow, roughly according to Poiseuille’s law.
4. ${\rm{\Delta }}p > 3.62\,g\cdot c{m}^{-1}\cdot {s}^{-2}$: When the favorable pressure gradient is large enough, the vessel no longer contracts because large amounts of NO are produced. Flow follows Poiseuille’s law in this regime.

## Discussion

In summary, we have developed a mathematical model based on tissue mechanics, mecho-sensitive feedback and lymph fluid dynamics that reproduces self sustained cyclic lymphatic contractions and fluid clearance from tissue. To optimize the model, we introduced valves with variable rigidity that are stiffer at the base than the tip. This is consistent with electron microscopic observations that the density of fibers is increased at the valve base^71^. For the fluid dynamics between the leaflets, it was necessary to incorporate lubrication forces, a simple and effective method to estimate forces when two moving fluid boundaries come into close proximity and there is no fluid node between them.

Our simulations reveal a number of interesting implications of the interplay between lymphatic valve structure and mechanobiological feedback control of contractions. Because of the inhibitory effect of NO on contractions, and the fact that its release is dependent on shear stresses, its spatiotemporal dynamics along the vessel wall –especially around valves and during backflow – can affect the propagation of contractions in interesting ways. In actual lymphatic vessels, the diameter is smallest at the valve^56,72^, and the shear stress is correspondingly higher in this region. Two other considerations tend to increase NO production at the valve leaflets: first, valve motion results in constantly changing shear stresses, which can increase NO release^73,74^ and second, fluid circulation between the valve leaflet and vessel wall means that NO can be released from both sides of the leaflet structure and become concentrated in this area. Thus, the valve performance (e.g., rest position and amount of leakage allowed) can determine lymph flow rates and the apparent direction of contraction propagation. Although the one-way valves ensure positive flow for any sequence of contractions, experimental observations conclude that the contractions can propagate either forward or retrograde with respect to the flow direction^18,19^. The reasons for this were previously unknown. We previously reported that if NO dominates, the contraction propagates retrograde, but if ${{\rm{Ca}}}^{2+}$ dominates, the contraction propagates forward^15^. Our current results with explicit mechanical valves extend this result, showing that the onset of contraction depends on the NO distribution during diastole. Many factors can shift the NO distribution along the lymphangion during diastole, including valve leakage, the double-sided nature of the valve leaflets and the pressure at the outlet. As these vary, the NO distribution can change, and the contractions initiate wherever NO is lowest. More importantly, these considerations can change the efficiency of fluid or cell transport through the lymphatic system.

A limitation of the model is that we have only a single lymphangion. Nevertheless, it is possible to observe at which end of the vessel the contractions initiate and to determine overall direction of propagation. Another limitation is that the lymphangion has to be anchored to fixed walls at the left and right to establish the boundary conditions. This limits motion of the valve structures somewhat compared to what is seen *in vivo*. In addition, we set the NO concentration at the boundaries to zero. However, *in vivo* we would expect there to be dynamic changes in the NO levels in nearby tissue contributed by other nearby lymphatic elements, including upstream and downstream. Future model development will include multiple lymphangions in series, an improvement that will address all these current limitations.

## Supplementary information

Supplementary video 1

Supplementary video 2

Supplementary video 3
